# Toy Models of Top Down Causation

**DOI:** 10.3390/e22111224

**Published:** 2020-10-27

**Authors:** Adrian Kent

**Affiliations:** 1 Centre for Quantum Information and Foundations, DAMTP, Centre for Mathematical Sciences, University of Cambridge, Wilberforce Road, Cambridge CB3 0WA, UK; A.P.A.Kent@damtp.cam.ac.uk; 2 Perimeter Institute for Theoretical Physics, 31 Caroline Street North, Waterloo, ON N2L 2Y5, Canada

**Keywords:** top-down causation, consciousness, panprotopsychism, cellular automata

## Abstract

Models in which causation arises from higher level structures as well as from microdynamics may be relevant to unifying quantum theory with classical physics or general relativity. They also give a way of defining a form of panprotopsychist property dualism, in which consciousness and material physics causally affect one another. I describe probabilistic toy models based on cellular automata that illustrate possibilities and difficulties with these ideas.

## 1. Introduction

The reductionist paradigm for theoretical physics suggests that the properties of complex structures, including their dynamics, can be understood as a consequence of their elementary components. It is not easy to characterise precisely what this means in all cases. Is a space–time or the vacuum state of a quantum field a complex structure, for example? And if so, what are their elementary components? Is a bare quark an elementary component or a mathematical fiction? Is quantum entanglement a counter-example to reductionism or just an illustration that the concept needs to be framed more carefully?

Nonetheless, it is widely agreed that some appropriately nuanced and qualified version of reductionism has been extremely successful, so much so that many theorists seek unified theories in which all of physics is characterised by some theory of the initial conditions together with relatively simple (though seemingly probabilistic) dynamical laws.

We should distinguish this strong but quite arguable stance from the stronger and fairly indefensible one that understanding the fundamental laws is the only really important task in science. As Anderson compellingly argued in his classic essay [1], solid-state physics, chemistry, biology, psychology and other higher-level theories produce new behaviours and new laws that require great inspiration and creativity to find and understand. But Anderson was nonetheless a card-carrying reductionist:

*The reductionist hypothesis may still be a topic for controversy among philosophers, but among the great majority of active scientists I think it is accepted without question. The workings of our minds and bodies, and of all the animate or inanimate matter of which we have any detailed knowledge, are assumed to be controlled by the same set of fundamental laws, which except under certain extreme conditions we feel we know pretty well.* [1]

Chalmers’ [2] distinction between types of emergence is very helpful here. High-level phenomena are *weakly* emergent when they are unexpected, but deducible in principle (even if not currently in practice) from fundamental theories. They are *strongly* emergent if they are not deducible even in principle. Reductionists aim for a relatively simple universal theory in which there are no examples of strong emergence, but should be comfortable with weak emergence. A representative survey would be very interesting, but my guess is that Anderson’s characterisation still holds true today: most scientists believe we already know enough of the fundamental theory to understand non-extreme regimes, and in particular would deny that the emergence of (quasi-)classicality from quantum theory, or (pace Chalmers [2,3]) of consciousness from classical or quantum physics, are clear examples of strong emergence. On the other hand, these questions are hotly debated among scientists working on consciousness and on quantum foundations.

Although the boundaries of reductionism may be slightly fuzzy, we can certainly produce models or theories that are clearly beyond them and unambiguously anti-reductionist. One example would be a theory that predicts qualitatively different dynamical equations for different types of molecule. (For the theory to be unambiguously anti-reductionist, it should not be possible to derive these equations from some simpler unifying principle. For example, dynamical collapse models [4,5] are not anti-reductionist, although in a sense they predict different behaviours for molecules of low and high mass: these predictions all follow from the same stochastic differential equation.) Another would be a vitalist theory that predicts that living creatures disobey some conservation law of classical mechanics. The general consensus is that we should assign low priors to such theories, not only because reductionism has been successful, but also because reductionist theories tend to be more elegant, and aligning credence with elegance has also been a very successful scientific methodology.

There is, though, more serious interest in better motivated models that do not fit the current mainstream reductionist paradigm. Consciousness, the topic of this special issue, *seems* to give more than one causal narrative—mind and matter *seem* to affect both themselves and each other. Yet the causal effects of matter on matter also seem enough for a complete description of the material world: there is no physical evidence that the known fundamental laws of physics do not suffice to describe the behaviour of the brain. There are (controversial) mainstream reductionist stances on this (e.g., [6]), but also well-known (also controversial) arguments (e.g., [3,7,8,9,10]) against the reducibility of consciousness to known physics. There has been an upsurge of interest lately in exploring alternative ideas involving new (or non-standard) physical hypotheses (e.g., [11,12,13,14,15,16,17,18,19]). Several of these have drawn inspiration and motivation from work on “integrated information theory” (IIT) [20] which, although open to many criticisms (e.g., [21,22,23], gives a mathematical framework to explore and generalise as well as a connection to empirical data.

Ideas of top-down causation have been mooted in the context of quantum theory and more broadly (e.g., [24,25]). The scope for top–down causal models of consciousness has not been extensively explored, and even the meaning and scope of top-down causation is not fully elaborated. This paper aims to give one framework for discussion, by describing some simple toy models, inspired by cellular automata, which illustrate possible ways in which higher level structures act causally on the microscopic components, as well as vice versa. It should be stressed that these are not meant to capture most realistic features of the world. The aim is to illustrate scope for more realistic models that use a similar mechanism to combine types of causation.

## 2. Cellular Automaton 110

Our toy models are based on cellular automata, but are not meant in the spirit of the well-known research programmes aiming to describe nature at a fundamental level in terms of cellular automata [26,27]. We use cellular automata simply as convenient illustrations.

Wolfram [26,28] classified the 256 elementary one-dimensional cellular automata. These are defined by binary states with a time step rule in which the states of cell *n* at time *t* are determined by those of cells n−1,n,n+1 at time (t−1). He noted the particularly interesting and complex behaviour of the automaton defined by rule 110 in his classification. Again, we pick this out not out of any fundamental preference—higher-dimensional cellular automata such the Game of Life [29] could equally well be used, for example—but for simplicity of illustration.

**Figure 1:**
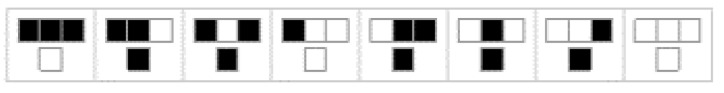
The rule 110 cellular automaton. The states of cells n−1,n,n+1 at time (t−1), given on the first row, determine that of cell *n* at time *t*, given on the second row.

Rule 110 is defined in Figure 1.

Wolfram had previously suggested [30] that rule 110 is Turing complete, a result subsequently proved by Cook [31]; another discussion of the result is given in [26]. We review here some of its known properties, using results and images generated by Wolfram Mathematica and resources [32] from the Wolfram Data Repository, which helpfully includes routines that reproduce many interesting diagrams originally given in [26].

**Figure 2:**
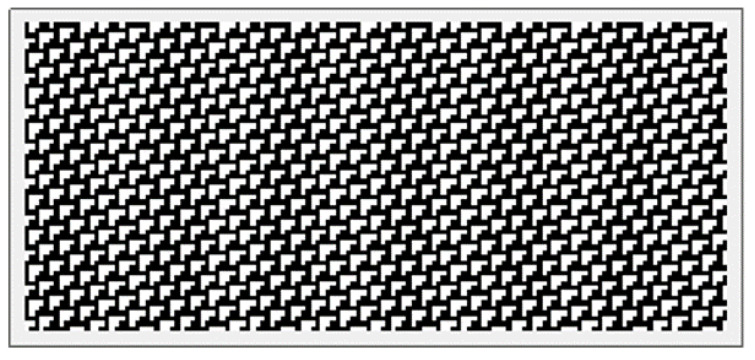
The ether.

The rule generates a regular repeating array with period 14, known as the “ether” as shown in Figure 2:

We will take this to be the analogue of a “vacuum” or “background state” in the toy models defined below.

**Figure 3:**
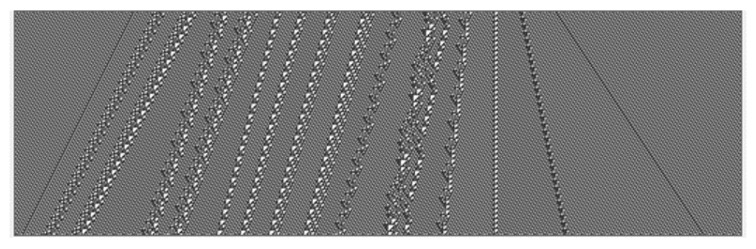
Gliders.

Some finite dislocations in the lattice-like 1D structure of a row of the ether can propagate regularly. These so-called “gliders” generate quasi-particle-like tracks in the ether. Cook [31] classified a variety of rule 110 gliders, including some infinite classes. Some of these are illustrated in Figure 3.

**Figure 4:**
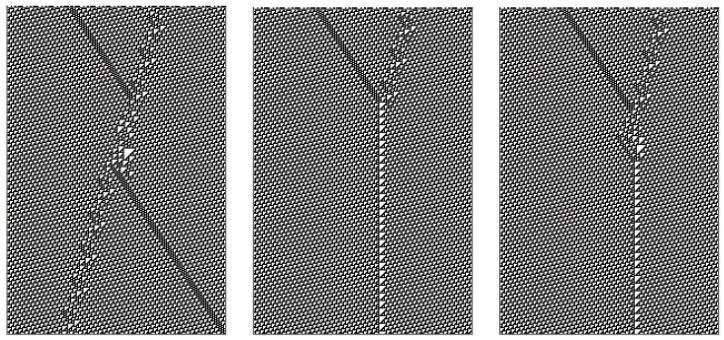
Interactions.

Colliding gliders undergo “interactions” that are superficially (of course, these are deterministic classical interactions, not complex quantum interaction amplitudes) reminiscent of Feynman diagrams, typically producing a finite number of stable new gliders after a finite interaction time: (For more discussion of the general phenomena of glider “particles” and background “domains” in cellular automata, see e.g., [33,34,35,36].) Some simple interactions are illustrated in Figure 4.

We can define a very simple model of errors or noise in these structures by considering the possibility of a single bit flip on the first row. One might motivate this by supposing that there is something particular about the system at t=0 that makes errors or noise possible on the first row, and only that row, (or so much more likely that we can neglect the possibility of errors on other rows) with error probability low enough that we can neglect the possibility of two or more errors arising.

**Figure 5:**
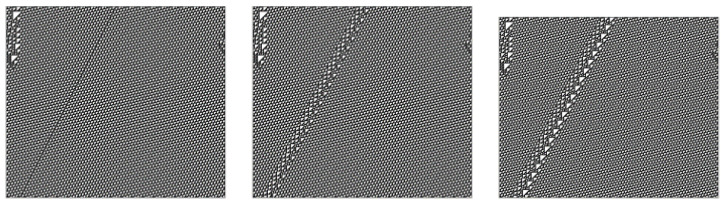
Perturbing the ether at a point distant from a glider.

If we consider a glider propagating in the ether, with a single bit of the initial state flipped at a site in the ether that is far from the glider, the effect tends to be simply to cause some ripples in the ether that propagate for some time and then peter out without interacting with the glider. The glider’s propagation is thus unaffected, as Figure 5 illustrates.

**Figure 6:**
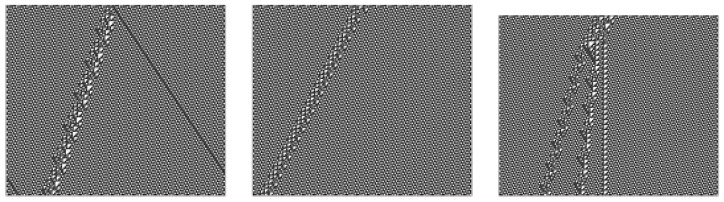
Perturbing the ether at a point close to a glider.

However, if we flip a bit close to a glider, it can interact in a way that permanently alters the number and type of gliders. Figure 6 shows the same glider states with one initial bit flipped. Only the second is asymptotically unaffected.

Perturbations affect interacting gliders similarly. A perturbation distant from interacting gliders generally peters out without affecting them. However, perturbations in the vicinity of one or more interacting gliders may alter the types and/or number of gliders in the final or asymptotic state.

**Figure 7:**
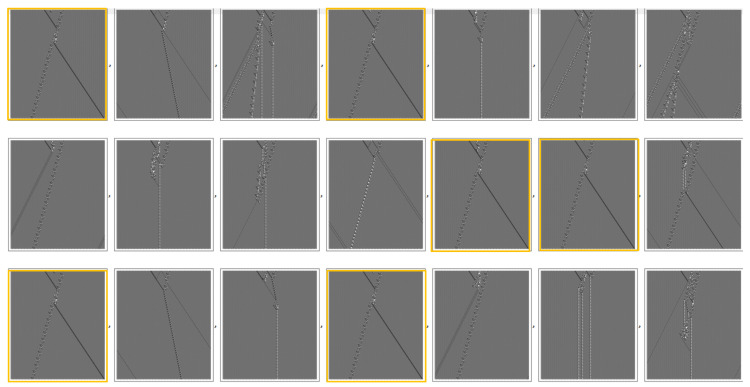
Perturbations near interacting gliders. The highlighted examples leave the final states asymptotically unchanged.

Figure 7 shows a pair of interacting gliders with a single flipped bit, whose site runs sequentially through 21 sites initially located between the gliders. Of these perturbations, the 1st, 4th, 12th, 13th, 15th and 18th leave the final glider states, highlighted, are asymptotically unchanged.

**Figure 8:**
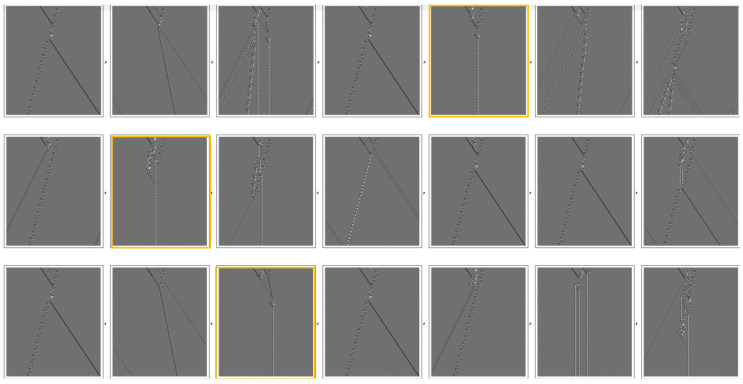
Perturbations near interacting gliders. The highlighted examples produce the same asymptotic final state, a single glider.

The 5th, 9th and 17th, highlighted in Figure 8, all produce the same new asymptotic final state, consisting of a single glider.

## 3. Probabilistic Models Based on Cellular Automata and Top-Down Causation

### 3.1. A Simple Probabilistic Model

We can formalise the model above as a 1D cellular automaton whose states are defined on sites labelled by the integers, at times also labelled by the integers. When the error probability *p* is zero, it is a deterministic type 110 automaton. The ether, or the ether with a single glider, then propagate indefinitely without perturbation. A pair of gliders may approach one another from infinity, interact and produce some number of outgoing gliders, which then propagate indefinitely.

We may also take the model to have finitely many spatial sites, with periodic boundary conditions. In this case, with appropriate numbers of sites, the ether and a single glider state may still propagate indefinitely. If a pair of gliders with different velocities are evident during some time interval, they will, so to speak, interact in both the past and future. If the interaction products contain two or more gliders with different velocities, they in turn will interact and the asymptotic behaviour may be quite complex. We can avoid this by defining the model only for a finite time interval, short compared to N/v, where *N* is the number of sites and *v* the maximum glider speed.

We suppose that there is some probability p>0 of an error occurring on the row of sites at t=0. An error flips the bit value of a single site, so that it takes the opposite value to that predicted by the deterministic dynamics from the state at t=−1. To simplify, we suppose that there is no probability of more than one error, and that errors are restricted to sites *x*, where −M≤x≤M, where (2M+1)≤N if there are finitely many (*N*) sites. The errors in this region have uniform probability, so that each site in the region has error probability $\frac{p}{2M+1}$.

The discussion of the previous section then applies: errors sufficiently far from any gliders at t=0 will typically peter out before interacting and have no effect on the final or asymptotic late time glider states; errors close to gliders can alter the number and type of final or asymptotic late time gliders.

### 3.2. Incorporating Top–Down Causation

We now consider modifying the dynamics by assigning probability weight factors to final glider states conditional on initial glider states.

One simple rule is to assign probability weight factor 1 to final states that are the same as the initial state for single glider propagation and two glider interactions, and weight factor 0 to distinct states. Formally,

(1) $$\begin{array}{ccc} p_{\mathrm{mod}}\left(G_{f}|G_{i}\right) & = & Cw\left(G_{f}|G_{i}\right)p\left(G_{f}|G_{i}\right)\;, \end{array}$$

(2) $$\begin{array}{ccc} p_{\mathrm{mod}}\left(G_{f}^{1},G_{f}^{2},\ldots ,G_{f}^{n}|G_{i}\right) & = & 0\;\;\mathrm{for}\;n\neq 1\;, \end{array}$$

(3) $$\begin{array}{ccc} p_{\mathrm{mod}}\left(G_{f}^{1},G_{f}^{2}|G_{i}^{1},G_{i}^{2}\right) & = & C^{\prime }w\left(G_{f}^{1},G_{f}^{2}|G_{i}^{1},G_{i}^{2}\right)p\left(G_{f}^{1},G_{f}^{2}|G_{i}^{1},G_{i}^{2}\right)\;, \end{array}$$

(4) $$\begin{array}{ccc} p_{\mathrm{mod}}\left(G_{f}^{1},G_{f}^{2},\ldots ,G_{f}^{n}|G_{i}^{1},G_{i}^{2}\right) & = & 0\;\;\mathrm{for}\;n\neq 2\;. \end{array}$$

Here

(5) $$w\left(G_{f}|G_{i}\right)=\delta_{G_{f},G_{i}}\;.$$

Multiple glider states are listed from left to right and so

(6) $$w\left(G_{f}^{1},G_{f}^{2}|G_{i}^{1},G_{i}^{2}\right)=\delta_{G_{f}^{1},G_{i}^{2}}\delta_{G_{f}^{2},G_{i}^{1}}\;$$

for colliding gliders, while

(7) $$w\left(G_{f}^{1},G_{f}^{2}|G_{i}^{1},G_{i}^{2}\right)=\delta_{G_{f}^{1},G_{i}^{1}}\delta_{G_{f}^{2},G_{i}^{2}}\;$$

for gliders that never collide. The expression $p(G_{f}|G_{i})$ is the probability of the final state containing (only) the single glider $G_{f}$ when the initial state contains glider $G_{i}$ in the model of the last subsection; $p(G_{f}^{1},G_{f}^{2}|G_{i}^{1},G_{i}^{2})$ is the probability of the final state containing (precisely) the pair $G_{f}^{1},G_{f}^{2}$ when the initial state contains the pair $G_{i}^{1},G_{i}^{2}$; *C* and $C^{\prime }$ are rescaling factors chosen so that the probabilities of all possible final states sum to 1 for a given initial state.

This rule is understood as applying to the system as a whole. It does not alter the deterministic dynamics of rule 110, and so its effect is to alter the probabilities of errors in the state at t=0, which are the only probabilistic feature of the toy model. For example, for an initial state containing a single glider *G*, it slightly increases the probability of errors at sites (such as those far from the glider) where they do not affect the glider propagation, increases the probability of no error and eliminates the possibility of errors occurring at sites where they would alter the asymptotic glider propagation. It has similar effects for initial states containing two gliders *G* and $G^{\prime }$. Effectively, the rule acts to suppress errors in glider propagation, ensuring the stability of one and two glider states, which would otherwise be menaced by possible errors in the microdynamics.

A variation is to assign probability weight 1 to specified final state outcomes of two glider interactions, and 0 otherwise, while retaining the weights above for single glider states Thus

(8) $$w\left(G_{f}|G_{i}\right)=\delta_{G_{f},G_{i}}$$

as above but $w(G_{f}^{1},G_{f}^{2},\ldots ,G_{f}^{n}|G_{i}^{1},G_{i}^{2})$ may have a more general form. For example, we might take $w(G_{f}|G_{i}^{1},G_{i}^{2})=1$ for some specified final state $G_{f}$, and zero for all other final states. This ensures that initial glider states $G_{i}^{1},G_{i}^{2}$ always produce final state $G_{f}$. However small the unmodified probability of this outcome is, so long as it is nonzero.

### 3.3. Compatibility with Standard Temporal and Minkowski Causation

Framed as above, these modified toy models may appear to involve something like instantaneous action at a distance, since the probability of error at a given site at t=0 effectively depends on the type and number of gliders at distant sites at the same time. If we think of the models as capturing the behaviour of particles (modelled by gliders) propagating in a background (the ether) with stochastic fluctuations (the errors), in some non-relativistic limit of a theory in relativistic space–time, this may seem to involve retrocausation: the probability of an error at a site depends on the final glider states in regions in its causal future.

While the models certainly could represent features of theories with non-standard causation, they are compatible with standard causation, even for relativistic theories. We can take the relevant glider speeds to be below light speed in such theories. The gliders contained in the state at t=0 depend deterministically on those contained in the states at t<0. We can thus equally well understand the probability of error of any site at t=0 as determined by the glider states at suitably large negative *t*, when the gliders are within the site’s past light cone. Interpreted in this way, errors at t=0 are causally determined by glider states at large negative *t*, according to laws that ensure specific glider states at large positive *t*. For example, one might imagine the models as capturing essential features of some deeper theory in which this causal determination is made more explicit, by degrees of freedom that carry information away non-superluminally from negative time glider states to sites throughout the ether and influence the error probabilities at t=0 appropriately.

## 4. Discussion

### 4.1. Panprotopsychist Models of Consciousness

There are reasons to consider physical models of consciousness that feature top down causation (although, as with every approach to consciousness, there are also problems and counterarguments). One line of argument runs as follows. There are evidently physical correlates of consciousness, namely human brains. If there is a fundamental physical law associating conscious states to physical systems, it seems unlikely that it associates consciousness to brains and to nothing simpler: brains seem too complex as physical systems to be the elementary referents of such a law. Full-blown panpsychism, in which every elementary particle has an associated elementary consciousness, is a possible alternative, but comes with many problems [37,38] and does not seem to fit naturally with neuroscientific data and our conscious self-observations or those reported by others. The intermediate option of panprotopsychism [39], according to which elementary consciousness is associated with some physical systems (whose nature remains to be specified) larger than elementary particles and smaller than brains, shares some of the problems of panpsychism, but allows more possibilities that might fit with empirical observation. Taking panprotopsychism seriously means accepting *some* sort of new physical law(s) associating the relevant systems with consciousness.

Our probabilistic models based on cellular automata can be taken as toy models of interactionist panprotopsychism. In these toy models, the elementary bits at each site are meant to correspond to elementary components, and the deterministic dynamics of rule 110 and unmodified error probability rules correspond to the elementary microdynamics. The gliders represent physical systems associated with elements of consciousness, which we might take to be quales or (if we stretch the present models even more unrealistically in order to illustrate how the idea might be extended) thoughts that we can represent by a sentence such as “I see blue”. The first modified versions of the model, in which the error probabilities are redefined to ensure that single gliders and pairs of gliders propagate unaffected by errors, then correspond to toy models in which panprotopsychist consciousness ensures error suppression at the level of consciousness, in the sense that quales (or thoughts) propagate unaffected in the substrate, despite errors in the microdynamics. The second modified versions redefine the error probabilities to ensure that pairs of gliders produce specified outcomes that would not arise in the absence of errors. These correspond to toy models in which panprotopsychist consciousness is equipped with its own dynamics, which overrides the dynamics of the substrate when the two conflict.

An argument in favour of something like this picture is that, if particular physical structures are indeed singled out as having an elementary proto-consciousness by fundamental physical laws, it is arguably natural that these physical structures should also feature in the fundamental dynamical laws. One might even speculate that nature has probabilistic laws because of the need to combine dual causalities, of matter and mind.

It is helpful to compare the pros and cons of this line of thought with those of a similarly panprotopsychist form of epiphenomenalism. This would associate consciousness in a lawlike way to specified physical structures, without modifying the microdynamics. The problem with this and other types of epiphenomenalism, as William James first pointed out [7], is that they leave all the apparently evolutionarily adaptive properties of consciousness unexplained. If physical laws of consciousness do not affect the microdynamics, then we and other creatures would function equally well if we were unconscious zombies, or if pleasure and pain were uncorrelated with evolutionary advantage, or if our consciousnesses were focussed on information that had no relevance to our survival or well-being, or if we had “locked-in” consciousnesses disconnected from any of our communications. On this view, we have to accept that not only the existence of consciousness, but the apparent fine-tuning of its specific features, are just astonishingly convenient coincidences.

In contrast, there is scope for more convincing explanations of the evolution of consciousness and of some of its features if dynamics give it a genuinely causal role in behaviour. It seems plausible, for example, that effectively coupling two types of dynamical rule allows evolution to more easily produce stimulus–response circuits that are more stable or follow higher-level reasoning. It also seems plausible that evolution would use this coupling to allow creatures to communicate their conscious states to one another. This would allow them to coordinate their behaviour better than communications that are influenced only by the microdynamics of their physical substrates, since in these models their behaviour may be directly affected by their conscious states. Models in which consciousness acts causally, via laws involving its complex physical correlates, also seem to offer some scope for explaining the correlation of pleasure (pain) with evolutionary (dis)advantage. A pain is something a conscious mind attempts to avoid, arguably by definition, and if the dynamics of conscious states reflect this, then evolution could naturally exploit this dynamics if disadvantageous physical situations created physical (brain) states that involved subsystems associated (via the hypothesized laws) with avoidant conscious states.

Even if these arguments can be made convincing, it would still seem a surprising and fortunate coincidence that, somewhere in the evolutionary chain, and perhaps very early, life took a material form that had proto-consciousness, and that matter and consciousness were associated in such a way that evolution was able to make use of the dynamical rules that give consciousness causal effect (via its material correlates) on matter. A priori, one might imagine that, if there are simple laws of psychophysical parallelism and simple associated dynamical laws, they need have nothing to do with self-replicating chemicals or organic information processing systems. So it is fair to ask how much fine-tuning interactionist panprotopsychist theories could explain, and how much they would still leave unexplained. Still, a partial explanation is better than none, and we also need to be clear whether we could possibly hope for a fuller explanation given our present conceptual frameworks. After all, we are conscious. Anyone who finds conceivability arguments [3,40] persuasive has to accept this, and all the features of our consciousnesses, as marvellous yet contingent features of our universe. On this view, we might hope that relatively simple laws characterise our consciousness and explain its evolution, but we cannot hope for an argument that the laws must take the form they do.

Closer analysis of all these arguments would undoubtedly be valuable. It would also be interesting to develop more sophisticated toy models, in which we can see rudimentary creatures evolving in a simple environment via modified dynamics.

### 4.2. Quantum Theory, Gravity and Classical Physics

As these toy models illustrate, probabilistic theories of microdynamics can be simply modified so that structures at two or more levels play roles in the fundamental laws. This makes it easy to build and explore models with top down causation. Such models could also potentially be relevant to unifying quantum theory and gravity. For example, one could imagine space–time emerging from a fundamentally quantum theory, within a theory in which it is equipped with its own independent dynamical laws; in such a theory, both space–time and its quantum constituents would causally affect one another, with neither reducible to the other. The same type of relationship is possible between classical and quantum degrees of freedom within a fixed background space–time. Classical physics is normally thought of as emerging from and reducible to quantum theory (by Everettians; see e.g., [41]) or some extension of quantum theory that does not radically alter the dynamics (by non-Everettians who believe some extension is needed to resolve the measurement problem). The latter looks plausible (e.g., [42,43]) and the simplest possibility, but it is interesting to ask how strongly empirical evidence constrains more general theories [43,44,45,46] that support this type of dual causation.
